# Supplementary material for: End Sequence Analysis Toolkit (ESAT) expands the extractable information from single-cell RNA-seq data
Source: Genome Res. 2016 Oct;26(10):1397–410. doi: 10.1101/gr.207902.116 (PMC5052061; doi:10.1101/gr.207902.116)
Supplement: Supplemental Material [file supp_gr.207902.116_Supp_Materials.tar.gz › ESAT-esat_v0.1/doc/html/classbroad_1_1core_1_1sequence_1_1_extractor.html]

ESAT: broad.core.sequence.Extractor Class Reference


ESAT


- Main Page
- Related Pages
- Packages
- Classes
- Files

- Class List
- Class Index
- Class Hierarchy
- Class Members

All Classes Namespaces Files Functions Variables Enumerator Pages

- broad
- core
- sequence
- Extractor

Public Member Functions |
Static Public Member Functions |
Static Public Attributes |
List of all members

broad.core.sequence.Extractor Class Reference

|  |  |
| --- | --- |
| Public Member Functions | |
|  | Extractor () |
|  | |

|  |  |
| --- | --- |
| Static Public Member Functions | |
| static void | main (String[] args) throws Exception |
|  | |

|  |  |
| --- | --- |
| Static Public Attributes | |
| static String | USAGE |
|  | |

## Constructor & Destructor Documentation

|  |  |  |  |  |
| --- | --- | --- | --- | --- |
| broad.core.sequence.Extractor.Extractor | ( |  | ) |  |

## Member Function Documentation

|  |  |  |  |  |  |
| --- | --- | --- | --- | --- | --- |
| static void broad.core.sequence.Extractor.main | ( | String[] | *args* | ) | throws Exception |

static

Here is the call graph for this function:

## Member Data Documentation

String broad.core.sequence.Extractor.USAGE

static

**Initial value:**

= "Usage: Extractor TASK=<task\_num> <task\_args>\n" +

"\tTasks:\n" +

"\t\t1. Batch Extract: IN=sequence id list file SEQ=sequence file OUT=output file\n" +

"\t\t2. Extract Regions: IN=sequence file OUT=output file REGIONS=start1..end1,start2..end2.... SEQID=sequence id if multifasta(use -eq- for = and -s- for spaces REVERSE=Y/N\n UPPERCASE=<Y to uppercase all letters>\n" +

"\t\t3. Extract records: -in <sequence file> -out <outout file> -seqids <seqId1,seqId2,seqId3,...> -reverse\n" +

"\t\t4. Extract and Chunk: IN=<sequence file> PREFIX=<output file prefix> CHUNKSIZE=<chunk size> OVERLAP=<chunk overlap> REGION=start1..end1 SEQID=sequence id if multifasta(use -eq- for = and -s- for spaces and -lp- parenthesis and -rp- for right parenthesis> REVERSE=<Y/N> UPPERCASE=<Y to uppercase all letters>\n" +

"\t\t5. Extract GC percent of all sequences in a multifasta file: IN=<sequence file>\n" +

"\t\t6. Unchunk Fasta chunks: INDIR=<Directory with sequence chunks it assumes chunks files are named seqname\_chunkStart\_chunkEnd.extension (specify file extension with the EXT parameter) > \n"+

"\t\t EXT=<chunk file extension> CHUNKSIZE=<chunk size> OVERLAP=<chunk overlap NOT HANDLING THIS YET>\n" +

"\t\t7. Extract records matching motif in reverse or direct strand -in <multifasta file> -motif <sequence motif> -out <output file name>\n" +

"\t\t8. Extract records with vClip preset parameters -in <multifasta>\n" +

"\t\t9. Extract GC percent for the specified window size: -in <sequence file> -out <GC percent outout file> -windowSize <window size>\n" +

"\t\t10. Reverse complement sequences in file: -in <sequence file or standard input> -out <file to output reverse-complemented sequences>" +

"\n\t\t11. Extract sequences that are of at least the given length: -in <sequence file or standard input> -out <file to output or standard out> + -minLength <Minumum length of sequences to return>" +

"\n\\t\t12. Extract sequences with names matching regular expression -in <sequence file or standard input> -out <file to output or standard out> -pattern <regex patterh>" +

"\n"

---

The documentation for this class was generated from the following file:

- java/broad/core/sequence/Extractor.java


---

Generated on Fri Aug 15 2014 15:10:55 for ESAT by  

 1.8.7
